# Supplementary material for: Resolving the full spectrum of human genome variation using Linked-Reads
Source: Genome Res. 2019 Apr;29(4):635–45. doi: 10.1101/gr.234443.118 (PMC6442396; doi:10.1101/gr.234443.118)
Supplement: Supplemental Material [file supp_gr.234443.118_Supplemental_longranger-2.2.zip › longranger-2.2.tar.bz2!/lr2.2-public/lib/stan/cmdstan/stan/doc/api/html/fwd_2numeric__limits_8hpp_source.html]

Stan: src/stan/agrad/fwd/numeric\_limits.hpp Source File


|  |  |
| --- | --- |
|  | Stan  2.5.0  probability, sampling & optimization |


- Main Page
- Related Pages
- Namespaces
- Classes
- Files

- File List
- File Members

All Classes Namespaces Files Functions Variables Typedefs Enumerations Enumerator Friends Macros Pages

- src
- stan
- agrad
- fwd

numeric\_limits.hpp

Go to the documentation of this file.

1 #ifndef STAN\_\_AGRAD\_\_FWD\_\_NUMERIC\_\_LIMITS\_\_HPP

2 #define STAN\_\_AGRAD\_\_FWD\_\_NUMERIC\_\_LIMITS\_\_HPP

3

4 #include <stan/agrad/fwd/fvar.hpp>

5 #include <stan/meta/traits.hpp>

6

7 namespace std {

8

9  template <typename T>

10

11  struct numeric\_limits<stan::agrad::fvar<T> > {

12  static const bool is\_specialized = true;

13  static stan::agrad::fvar<T> min() { return numeric\_limits<double>::min(); }

14  static stan::agrad::fvar<T> max() { return numeric\_limits<double>::max(); }

15  static const int digits = numeric\_limits<double>::digits;

16  static const int digits10 = numeric\_limits<double>::digits10;

17  static const bool is\_signed = numeric\_limits<double>::is\_signed;

18  static const bool is\_integer = numeric\_limits<double>::is\_integer;

19  static const bool is\_exact = numeric\_limits<double>::is\_exact;

20  static const int radix = numeric\_limits<double>::radix;

21  static stan::agrad::fvar<T> epsilon() {

22  return numeric\_limits<double>::epsilon(); }

23  static stan::agrad::fvar<T> round\_error() {

24  return numeric\_limits<double>::round\_error(); }

25

26  static const int min\_exponent = numeric\_limits<double>::min\_exponent;

27  static const int min\_exponent10 = numeric\_limits<double>::min\_exponent10;

28  static const int max\_exponent = numeric\_limits<double>::max\_exponent;

29  static const int max\_exponent10 = numeric\_limits<double>::max\_exponent10;

30

31  static const bool has\_infinity = numeric\_limits<double>::has\_infinity;

32  static const bool has\_quiet\_NaN = numeric\_limits<double>::has\_quiet\_NaN;

33  static const bool has\_signaling\_NaN =

34  numeric\_limits<double>::has\_signaling\_NaN;

35  static const float\_denorm\_style has\_denorm =

36  numeric\_limits<double>::has\_denorm;

37  static const bool has\_denorm\_loss = numeric\_limits<double>::has\_denorm\_loss;

38  static stan::agrad::fvar<T> infinity() {

39  return numeric\_limits<double>::infinity(); }

40  static stan::agrad::fvar<T> quiet\_NaN() {

41  return numeric\_limits<double>::quiet\_NaN(); }

42  static stan::agrad::fvar<T> signaling\_NaN() {

43  return numeric\_limits<double>::signaling\_NaN(); }

44  static stan::agrad::fvar<T> denorm\_min() {

45  return numeric\_limits<double>::denorm\_min(); }

46

47  static const bool is\_iec559 = numeric\_limits<double>::is\_iec559;

48  static const bool is\_bounded = numeric\_limits<double>::is\_bounded;

49  static const bool is\_modulo = numeric\_limits<double>::is\_modulo;

50

51  static const bool traps = numeric\_limits<double>::traps;

52  static const bool tinyness\_before = numeric\_limits<double>::tinyness\_before;

53  static const float\_round\_style round\_style =

54  numeric\_limits<double>::round\_style;

55  };

56 }

57 #endif

std::numeric\_limits< stan::agrad::fvar< T > >::max

static stan::agrad::fvar< T > max()

**Definition:** numeric\_limits.hpp:14

std::numeric\_limits< stan::agrad::fvar< T > >::signaling\_NaN

static stan::agrad::fvar< T > signaling\_NaN()

**Definition:** numeric\_limits.hpp:42

stan::agrad::fvar

**Definition:** fvar.hpp:13

std::numeric\_limits< stan::agrad::fvar< T > >::min

static stan::agrad::fvar< T > min()

**Definition:** numeric\_limits.hpp:13

stan::math::max

double max(const double a, const double b)

**Definition:** max.hpp:7

std::numeric\_limits< stan::agrad::fvar< T > >::infinity

static stan::agrad::fvar< T > infinity()

**Definition:** numeric\_limits.hpp:38

traits.hpp

std::numeric\_limits< stan::agrad::fvar< T > >::round\_error

static stan::agrad::fvar< T > round\_error()

**Definition:** numeric\_limits.hpp:23

fvar.hpp

std::numeric\_limits< stan::agrad::fvar< T > >::epsilon

static stan::agrad::fvar< T > epsilon()

**Definition:** numeric\_limits.hpp:21

std::numeric\_limits< stan::agrad::fvar< T > >::quiet\_NaN

static stan::agrad::fvar< T > quiet\_NaN()

**Definition:** numeric\_limits.hpp:40

std::numeric\_limits< stan::agrad::fvar< T > >::denorm\_min

static stan::agrad::fvar< T > denorm\_min()

**Definition:** numeric\_limits.hpp:44

stan::math::min

double min(const double a, const double b)

**Definition:** min.hpp:7


---

[ Stan Home Page ]

*© 2011–2014,
Stan Development Team.*
